# Invasive breast cancer induces laminin-332 upregulation and integrin β4 neoexpression in myofibroblasts to confer an anoikis-resistant phenotype during tissue remodeling

**DOI:** 10.1186/bcr3203

**Published:** 2012-06-06

**Authors:** Baek Gil Kim, Ming-Qing Gao, Yoon Pyo Choi, Suki Kang, Haeng Ran Park, Kyu Sub Kang, Nam Hoon Cho

**Affiliations:** 1 Brain Korea 21 Project for Medical Science, Yonsei University College of Medicine, 134 Sinchon-dong, Seodaemun-gu, Seoul, 120-752, South Korea; 2 Severance Biomedical Science Institute (SBSI), Yonsei University College of Medicine, 134 Sinchon-dong, Seodaemun-gu, Seoul, 120-752, South Korea; 3 Department of Pathology, Yonsei University College of Medicine, 134 Sinchon-dong, Seodaemun-gu, Seoul, 120-752, South Korea; 4 Global 5-5-10 System Biology, Yonsei University, 50 Yonsei-ro, Seodaemun-gu, Seoul, 120-749, South Korea

## Abstract

**Introduction:**

Although development of anoikis-resistant myofibroblasts during tissue remodeling is known to be associated with tumor invasion, the mechanism by which myofibroblasts become resistant to anoikis is unknown. We previously demonstrated laminin-332 upregulation in the fibrosis around invasive ductal carcinoma (IDC). Because laminin-332 promotes cell survival through binding to integrins, we hypothesized that invasive breast cancer cells confer an anoikis-resistant phenotype on myofibroblasts by upregulating laminin-332 expression during tissue remodeling. Here, we demonstrate that invasive breast cancer cells induce laminin-332 upregulation and integrin β4 neoexpression in myofibroblasts to confer an anoikis-resistant phenotype.

**Methods:**

Three types of fibroblasts were isolated from the tumor burden, the fibrosis, and normal tissue of patients with early stage IDC (less than 10 mm diameter), designated cancer-associated fibroblasts (CAFs), interface fibroblasts (InFs), and normal breast fibroblasts (NBFs), respectively. To investigate direct and indirect crosstalk with tumor cells, fibroblasts were co-cultured with invasive MDA-MB-231 or noninvasive MCF7 cells or in conditioned medium. Anoikis resistance of fibroblasts was measured by cell viability and caspase-3 activity after incubation on poly-HEMA coated plates for 72 hours. Involvement of laminin-332/integrin α3β1 or α6β4 signaling in anoikis resistance was confirmed by treatment with purified laminin-332 or blocking antibodies against laminin-332, integrin β1, or integrin β4.

**Results:**

MDA-MB-231 cells induced laminin-332 upregulation and integrin β4 neoexpression in fibroblasts, leading to anoikis resistance. InFs showed a higher endogenous level of laminin-332 than did CAFs and NBFs. After stimulation with MDA-MB-231-conditioned medium, laminin-332 expression of InFs was dramatically increased and maintained under anoikis conditions. Laminin-332 upregulation was also observed in CAFs and NBFs, but at a lower level than in InFs. Laminin-332 induced Akt (Ser473) phosphorylation by binding to integrin α3β1. Integrin β4 neoexpression induced laminin-332-independent Rac1 activation and promoted anoikis resistance in fibroblasts approximately twofold more effectively than did laminin-332, regardless of the type of fibroblast. In addition, integrin β4 expression suppressed fibroblast aggregation in conditions of anoikis.

**Conclusion:**

Invasive breast cancer cells confer an anoikis-resistant phenotype on myofibroblasts during tissue remodeling by inducing laminin-332 upregulation and integrin β4 neoexpression. Interface fibroblasts appear to be the primary myofibroblasts that interact with invasive tumor cells during tissue remodeling.

## Introduction

A fundamental component of tumor invasion is stromal tissue remodeling, which involves proteolytic degradation of the extracellular matrix (ECM) and results in anoikis (a form of caspase-dependent apoptosis that is caused by loss of integrin binding of stromal cells) [1]. As a component of stroma, myofibroblasts are also exposed to anoikis during tissue remodeling. However, many studies have reported prolonged survival of myofibroblasts during tissue remodeling in patients with fibrotic diseases [2-5]. Fibrosis is considered an indicator of tissue remodeling [6] and is commonly formed around invasive types of tumors [7-9]. Considering that myofibroblasts are key regulators of tissue remodeling [10] and a major source of ECM production [11], which drives tumor progression, the development of anoikis-resistant myofibroblasts may be an essential event during stromal tissue remodeling before tumor invasion.

Abnormal and excessive ECM deposition not only is a phenotype of fibrosis but also is associated with cell-survival signaling mediated by integrin receptors during tumor invasion and tissue remodeling [12]. Therefore, altered molecular expression in fibrosis may provide a clue to how myofibroblasts acquire an anoikis-resistant phenotype during tissue remodeling. Previously, we observed aberrant laminin-332 upregulation in the fibrosis of invasive ductal carcinoma (IDC) compared with autologous tumor burden and distal normal tissue, whereas such laminin-332 upregulation was not found in the noninvasive counterpart, ductal carcinoma *in situ *(DCIS) [13]. Contrary to our finding, laminin-332 expression was previously reported to be downregulated in breast cancer [14]. Laminin-332, a large multidomain molecule involved in cell adhesion and matrix assembly, plays an important role in cell migration and survival by activating many signal mediators through binding to integrin α3β1 or α6β4 [15-18]. The role of laminin-332 in cell survival or anoikis resistance has been studied mostly in epithelial tumor cells, although some data exist on laminin-332-dependent survival of keratinocytes during wound healing [19,20]. One group demonstrated that the matricellular protein thrombospondin1 (TSP1) induces anoikis resistance in mouse embryonic fibroblasts *in vitro *[21]. This work provided convincing evidence that an ECM protein can induce anoikis resistance in fibroblasts and suggested that laminin-332 may fulfill a similar function. However, because the fibroblasts used in previous studies were not derived from tumor stroma, the role of anoikis resistance of myofibroblasts in the context of tumor invasion remains undetermined.

In this study, we hypothesized that invasive breast cancer cells induce laminin-332 upregulation in myofibroblasts undergoing tissue remodeling, which leads to anoikis resistance through laminin-332 autocrine signaling. To test our hypothesis, we used three types of fibroblasts isolated from IDC tissue and co-cultured them with breast cancer cells to investigate tumor-stroma crosstalk. Our results provide evidence that anoikis-resistant myofibroblasts develop during tissue remodeling of invasive breast cancer as a result of abnormal upregulation of ECM proteins and/or their receptors.

## Materials and methods

### Isolation of primary fibroblasts

Human breast tumors and autologous normal breast tissues were obtained from three IDC patients undergoing surgery at Severance Hospital of the Yonsei University Health System, Korea. The protocol for the study was approved by Severance Hospital Ethics Committee (4-2008-0380). All participants signed written informed consent forms detailing tissue use for comprehensive experiments on breast cancer. Based on the zonal concept proposed in our previous study, primary fibroblasts were isolated from tissue in the tumor zone (TZ, tumor burden), interface zone (IF, fibrosis), and normal zone (distal normal tissue), as described previously [22].

### Direct and indirect co-culture

For direct co-culture, primary fibroblasts were cultured with MCF7 or MDA-MB-231 breast cancer cells (Korean Cell Line Bank, Seoul, Korea), as described previously [23]. MDA-MB-231 and MCF7 cells were selected as representative invasive and noninvasive types of breast cancer, respectively. Adherent fibroblasts were stained with 5 μ*M *Cell Tracker Green CMFDA (5-chloromethylfluorescein diacetate; Invitrogen, Eugene, OR, USA) by incubation at 37°C for 45 minutes. The dye solution was then replaced with fresh prewarmed medium, and the cells were incubated at 37°C for another 2 hours. After washing them twice with PBS, unstained cancer cells were plated onto a layer of CMFDA-stained fibroblasts. The co-cultures were incubated in reduced serum media (RSM) composed of DMEM/F12, 1% FBS, 100 IU/ml penicillin, and 100 μg/ml streptomycin for 1 week. CMFDA-stained fibroblasts and unstained breast cancer cells were sorted with flow cytometry and used for subsequent experiments. For indirect co-culture, conditioned medium (CM) was prepared by culturing MCF7 or MDA-MB-231 breast cancer cells at 80% confluency in RSM for 1 day. The CM was filtered through a 0.22-μm filter (Millipore, Billerica, MA, USA) and diluted 1:1 with fresh RSM before addition to fibroblast cultures, which were incubated for a further 3 days.

### Transfection of fibroblasts with integrin β4

For transient transfection with integrin β4, fibroblasts (5 × 10^5^ cells/well) were plated on a six-well plate and transfected with 3 μg pRK6 b4 plasmid DNA (Addgene plasmid 16037; Addgene, Cambridge, MA, USA; kindly donated by Dr. FG Giancotti, Cellular Biochemistry and Biophysics Program, Memorial Sloan-Kettering Cancer Center, New York, NY, USA) [24], and 3 μl MATRA (Promokine, Heidelberg, Germany) in a total volume of 200 μl Opti-MEM on a Universal Magnet Plate (Promokine) for 15 minutes. Controls were transfected with 3 μg pRK5.

### Anoikis assays

Fibroblasts (5 × 10^5^ cells/well in 1 ml) were added to either poly-HEMA- or serum-coated wells and incubated at 37°C in 5% CO_2_ with RSM, MCF-7 CM, or MDA-MB-231 CM for 6, 24, 48, or 72 hour. Maintenance of suspended fibroblasts in poly-HEMA-coated wells (anoikis conditions) was monitored by phase microscopy. Cell viability was measured by addition of 100 µl CCK-8 (Dojindo Molecular Technologies, Inc., Rockville, MD, USA) to each well. After incubation at 37°C for 2 hours, fluorescence was measured with a plate reader (excitation, 530 nm/emission, 580 nm). Fibroblasts from three IDC patients were assayed in triplicate in at least three separate experiments. For function-blocking assays, antibodies against integrin β1, β4, or laminin-332 (Millipore) were added to InF cells (which have high expression of laminin-332) with MDA-MB-231 CM and incubated at 37°C in a humidified 5% CO_2 _incubator for 24 hours. CAF cells (which express low levels of laminin-332) were treated with purified laminin-332 (Abcam, Cambridge, England) in RSM for 24 hours.

### Caspase-3 activity assay

Caspase-3 activity in fibroblast lysates was measured by using a caspase-3 activity kit (Becton-Dickinson-Pharmingen, San Jose, CA, USA). Fibroblasts (5 × 10^5 ^cells) were plated in poly-HEMA-coated-wells with RSM, MCF7 CM, or MDA-MB-231 CM and treated with blocking antibodies or purified laminin-332 for 24 hours. Fibroblast lysates were incubated in a 96-well plate in HEPES buffer with 5 µl caspase-3 fluorogenic substrate (Ac-DEVD-AMC) for 1 hour at 37°C in the dark. Fluorescence was measured with a plate reader (excitation, 360 nm/emission, 460 nm).

### Rac1 pull-down assay

Suspended fibroblasts were harvested by centrifugation and lysed in 1× lysis buffer. Rac/cdc42 assay reagent (10 µl; 10 µg) was added to 0.5 ml cell lysate, and the reaction mixture was incubated by rocking gently at 4°C for 1 hour. Agarose beads were collected by pulsing for 5 seconds in a microcentrifuge at 14,000 *g*, and the supernatant was discarded. The pelleted beads were washed 3 times with 0.5 ml Mg^2+^ Lysis/Wash Buffer (MLB), resuspended in 40 µl 5× Laemmli reducing sample buffer, boiled for 5 minutes, separated by polyacrylamide gel electrophoresis (PAGE), and then detected with immunoblot.

### Immunoblotting

Suspended cells were collected by centrifugation, lysed in 100 μl Protein Extraction Solution (Intron Biotech, Seongnan-si, Korea), homogenized with a 30-gauge needle, incubated for 30minutes at 4°C, and then purified by centrifugation at 13,000 rpm. After quantifying proteins in the extracts by using the Bradford method, 20 μg protein was electrophoresed on 10% Tris/glycine gels (Invitrogen, Carlsbad, CA, USA), transferred to polyvinylidine fluoride (PVDF) membranes (Millipore), and probed with primary antibodies against laminin γ2, integrin α3, integrin α6, integrin β1, integrin β4, Akt, phospho(Ser473)-Akt, E-cadherin, β-catenin, GAPDH (Santa Cruz Biotechnology, Santa Cruz, CA, USA), or Rac1 (Millipore). Primary antibodies were detected with horseradish peroxidase (HRP)-conjugated secondary antibodies (Invitrogen), and visualized by using enhanced chemiluminescence reagents (Santa Cruz Biotechnology).

### Statistics

Statistical significance (indicated by asterisks) was determined by using the *t *test and ANOVA. Results were considered to be significant at *P *< 0.05. All statistical analyses were performed by using SPSS version 11.5 for Windows statistical software (SPSS Inc.).

## Results

### Invasive breast cancer cells induce upregulation of laminin-332 expression in stromal fibroblasts

**Figure 1:**
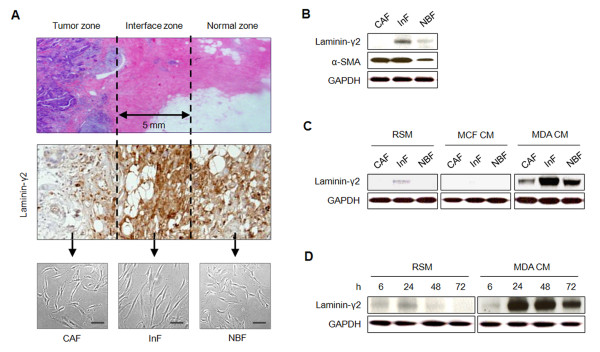
**Laminin-332 expression in stromal fibroblasts is upregulated by invasive breast cancer cells**. **(A) **Isolation of stromal fibroblasts from invasive ductal carcinoma (IDC). Based on the zonal concept introduced in our previous study, fibroblasts were isolated from early-stage IDC tissue (less than 10 mm diameter) by enzyme digestion after confirmation of laminin-γ2 expression. **(B) **Expression of laminin-γ2 and α-SMA in fibroblasts of IDC. **(C) **Stimulation of laminin-γ2 expression in fibroblasts by CM from cancer cells. Fibroblasts were incubated with RSM, MCF7 CM, or MDA-MB-231 CM for 24 hours. **(D) **Duration of laminin-γ2 expression in interface fibroblasts (InFs) under anoikis conditions. InFs (5 × 10^5 ^cell/well) were seeded onto poly-HEMA coated six-well plates with RSM or MDA-MB-231 CM for 72 hours. Suspended cells were collected at 6, 24, 48, and 72 hours, as indicated.

Based on the zonal concept that we previously introduced, stromal fibroblasts were isolated from each zone of IDC tissue after confirming expression of laminin-γ2, a subunit used to identify laminin-332. Primary fibroblasts from the tumor zone (TZ), interface zone (IZ), and normal zone were designated cancer-associated fibroblasts (CAFs), interface fibroblasts (InFs), and normal breast fibroblasts (NBFs), respectively. In morphology, InFs were slightly longer than CAFs and NBFs (Figure 1A). After culture, InFs expressed a higher level of laminin-γ2 than did CAFs and NBFs, as they do *in situ*, and InFs and CAFs expressed a higher level of α-SMA did than NBFs (Figure 1B). To test whether factors secreted by invasive breast cancer cells can further increase fibroblast laminin-γ2 expression, we treated cultured fibroblasts with MDA-MB-231 CM. MDA-MB-231 CM dramatically increased laminin-γ2 expression in InFs compared with CAFs and NBFs. Thus, although MDA-MB-231 CM enhanced laminin-γ2 expression in all fibroblasts, the degree of increase depended on the intrinsic capacity of the cells to express laminin-γ2. In contrast, MCF7 CM suppressed laminin-γ2 expression (Figure 1C). Because the IZ is densely fibrotic, a hallmark of the tissue remodeling that leads to anoikis of stromal cells, the capacity of InFs to express laminin-332 under anoikis conditions was evaluated in the absence or presence of MDA-MB-231 CM stimulation. In InFs stimulated with MDA-MB-231 CM, overexpression of laminin-γ2 was evident at 24 hours and was maintained for 72 hours (Figure 1D). Because transforming growth factor (TGF)-β is a major component of MDA-MB-231 CM, we speculated that it might be responsible for inducing laminin-332 overexpression. To test this, we treated InFs with 100 n*M *LY2157299, a TGF-β inhibitor, and MDA-MB-231 CM for 24 hours and found that laminin-γ2 expression was suppressed in the LY2157299-treated InFs (see Additional file 1, Figure S1).

### Integrin β4 neoexpression in fibroblasts is induced through direct contact with invasive breast cancer cells

**Figure 2:**
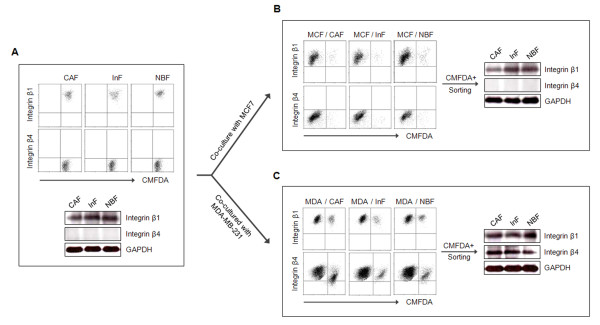
**Integrin β4 neoexpression is induced in fibroblasts through direct contact with invasive breast cancer cells**. **(A) **Endogenous expression of integrin β1 and β4 in fibroblasts of IDC. Fibroblasts were prestained with CMFDA and cultured in RSM, MCF7 CM, or MDA-MB-231 CM for 72 hours before staining with PE-conjugated anti-integrin β1 or β4 antibodies. **(B) **Integrin β1 expression and **(C) **Integrin β4 expression in fibroblasts cocultured with MCF7 or MDA-MB-231 cells. Confluent fibroblasts on 100-mm dishes were stained with 5 μ*M *CMFDA. MCF7 or MDA-MB-231 cells (5 × 10^5^) were added to the stained fibroblasts and cocultured for 1 week. Mixed cells were collected and stained with PE-conjugated anti-integrin β1 or β4 antibodies. Integrin β1 or β4 expression in cocultured fibroblasts was confirmed by immunoblot analysis after sorting.

Two integrin receptors for laminin-332, integrins α3β1 and α6β4, are associated with signaling pathways for cell survival. A high level of integrins α3 and α6 expression was found in the fibroblasts derived from IDC (see Additional file 1 Figure S2). Integrin β1 was also highly expressed in the fibroblasts, whereas integrin β4 was not expressed (Figure 2A). It is widely known that the repertoire or pattern of integrin expression in stromal cells can be changed through communication with tumor cells. Because the IZ is adjacent to the TZ within tissue, CAFs and InFs can directly or indirectly communicate with tumor cells, and, as a result, the pattern of integrin expression in myofibroblasts may be altered. To test the effect of indirect communication with tumor cells, the fibroblasts were stimulated with MDA-MB-231 CM or MCF7 CM. Integrin β1 expression was not significantly affected, but remained high, independent of CM stimulation (see Additional file 1, Figure S3A). Integrin β4 expression was also unchanged and remained undetectable (see Additional file 1, Figure S3B). For direct communication with tumor cells, fibroblasts were co-cultured with MDA-MB-231 or MCF7 cells. Integrin β1 expression was not affected by direct contact with cancer cells, but was highly sustained (Figure 2B). In contrast, *de novo *expression of integrin β4 was induced by direct contact with MDA-MB-231, but not MCF7, cells (Figure 2C). Neoexpression of integrin β4 protein was also detected in fibroblasts after sorting from the co-culture.

### Anoikis resistance of fibroblasts is conferred by binding of laminin-332 to integrin α3β1 and by neoexpression of integrin β4

**Figure 3:**
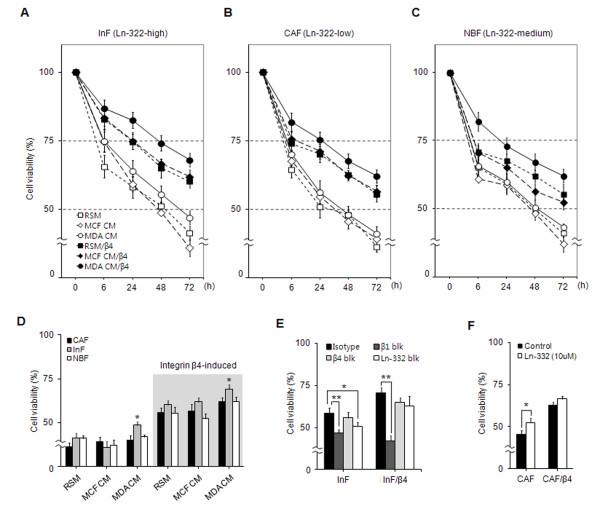
**The anoikis resistance of fibroblasts is mediated by binding of laminin-332 to integrin α3β1 and/or integrin β4 neoexpression**. Viability of wild-type and integrin β4-expressing **(A) **InFs, **(B) **CAFs, and **(C) **NBFs in the absence or presence of stimulation from cancer cells under anoikis conditions. The figures show fibroblast viability in RSM (open square), MCF7 CM (open diamond), and MDA-MB-231 CM (open circle) in the absence of integrin β4 expression, and viability in RSM (black square), MCF7 CM (black diamond), and MDA-MB-231 CM (black circle) in the presence of integrin β4 expression. **(D) **Direct comparison of fibroblast viabilities. The viability of wild-type and integrin β4-expressing fibroblasts was compared at 72 hours. Black bar, CAFs; gray bar, InFs; white bar, NBFs; gray box, integrin β4-expressing fibroblasts. **(E) **Inhibition of the interaction between laminin-332 and integrin. InF and InF/β4 cells were treated with isotype (black bar) and blocking antibodies against integrin β1 (dark gray bar), β4 (light gray bar), and laminin-332 (white bar) for 24 hours under anoikis conditions. **(F) **Increased viability of CAFs in response to treatment with laminin-332. CAF and CAF/β4 cells were treated with purified laminin-332 (diluted to 10 μ*M *with RSM) in poly-HEMA-treated 96-well plates for 24 hours. Black bar, untreated CAFs; white bar, laminin-322-treated CAFs. Results are expressed as mean ± SD. *P < 0.05; **P < 0.03 versus RSM in poly-HEMA-coated wells. Results are averages of three separate experiments.

We found that MDA-MB-231 cells induced laminin-332 upregulation and integrin β4 neoexpression in fibroblasts, and that the fibroblasts expressed a high level of integrin α3, α6, and β1 independent of their interaction with cancer cells (Figure 2, and see Additional file 1, Figure S2). Therefore, we hypothesized that the fibroblasts in IDC overcome anoikis through laminin-332-dependent survival pathways that are mediated by integrin α3β1 and/or α6β4. For the anoikis assay, laminin-332 upregulation in fibroblasts was induced by treatment with MDA-MB-231 CM, and integrin β4 neoexpression was induced by transfection with an integrin β4 plasmid. Integrin β4 overexpression was confirmed in all transfected fibroblasts (see Additional file 1, Figure S4). Anoikis resistance of fibroblasts was examined by measuring their viability under anoikis conditions. In the absence of integrin β4 expression, the viability of InFs (which express high levels of laminin-332) was increased by MDA-MB-231 CM but decreased by MCF7 CM, compared with the control incubated with reduced serum media (RSM) only. In the presence of integrin β4 expression, the viability of InFs was dramatically increased at 6 hours and maintained at a level approximately twofold higher than that without integrin β4 through 72 hours. In addition, the viability of these cells was further enhanced by treatment with MDA-MB-231 CM (Figure 3A). Similar to that of InFs, the viability of CAFs (which express low levels of laminin-332) was increased by MDA-MB-231 CM and integrin β4 induction, although the viability of CAFs was lower than that of InFs (Figure 3B). The viability of NBFs (expressing medium levels of laminin-322) showed a pattern similar to those of InFs and CAFs (Figure 3C). The viability of the three types of fibroblasts at 72 hours is directly compared in Figure 3D. Induction of integrin β4 enhanced the viability of all fibroblast types approximately twofold compared with fibroblasts lacking integrin β4. In contrast, MDA-MB-231 CM only increased the viability of InFs, and to a lesser extent than did integrin β4 induction.

To confirm that fibroblast viability was associated with binding of laminin-332 to integrins α3β1 and/or α6β4, InFs and InFs/β4 (expressing integrin β4) were first stimulated with MDA-MB-231 CM to induce laminin-332 upregulation and then treated with blocking antibodies against laminin-332, integrin β1, or integrin β4. The viability of both InF and InFs/β4 was inhibited by blocking integrin β1 and laminin-332, whereas blocking integrin β4 had little effect (Figure 3E).

To show that the viability of fibroblasts depends on laminin-332 expression, CAFs (Ln-332-low) were treated with 10 μ*M *purified laminin-332, resulting in increased viability. The viability of CAFs/β4 was strongly maintained, independent of laminin-332 treatment (Figure 3F).

### Laminin-332 upregulation and integrin β4 neoexpression suppress caspase-3 activity in fibroblasts

**Figure 4:**
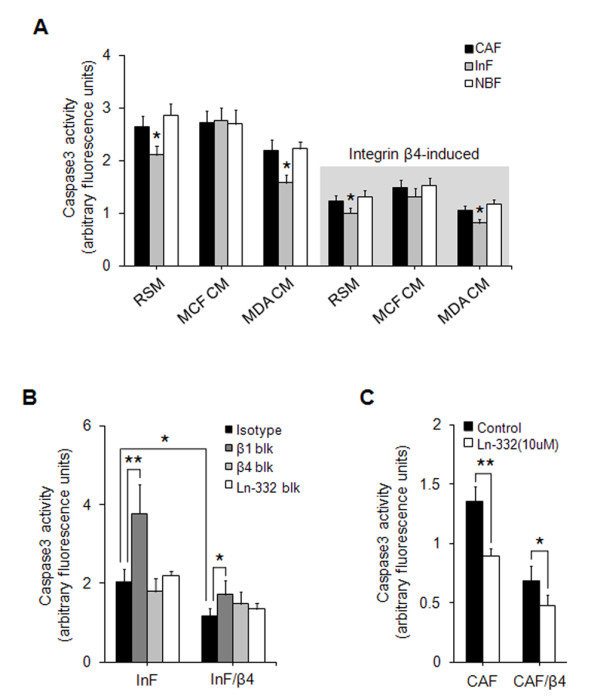
**Laminin-332 upregulation and integrin β4 neoexpression suppressed caspase-3 activity in fibroblasts**. **(A) **Caspase-3 activity of fibroblasts under anoikis conditions in the absence or presence of integrin β4 and/or stimulation with CM from cancer cells. Black bar, CAF; gray bar, InF; white bar, NBF; gray box, integrin β4-expressing fibroblasts. **(B) **Caspase-3 activity according to inhibition of integrin β1 (dark-gray bar), β4 (light-gray bar), or laminin-332 (white bar). The black bar shows the isotype control. **(C) **Effect of laminim-322 treatment on caspase-3 activity in CAFs. Black bar, untreated CAFs; white bar, laminin-322-treated CAFs. Results are expressed as mean ± SD. *P < 0.05; **P < 0.03 versus RSM in poly-HEMA-coated wells. Results are averages of three separate experiments.

Anoikis is a form of caspase-dependent apoptosis that is caused by loss of integrin binding. Because caspase-3 is a key effector in the extrinsic apoptotic pathway, we examined caspase-3 activity in fibroblasts in the absence or presence of cancer cell CM for 72 hours. Without integrin β4 expression, InFs had lower caspase-3 activity than did CAFs and NBFs, and MDA-MB-231 CM further reduced caspase-3 activity, whereas MCF7 CM had no effect. In addition, the induction of integrin β4 dramatically reduced caspase-3 activity, which also became much less dependent on MDA-MB-231 CM stimulation. The lowest measured caspase-3 activity was in InFs/β4 cells stimulated with MDA-MB-231 CM (Figure 4A). To confirm whether the caspase-3 activity of fibroblasts is regulated by the binding of laminin-332 to integrin α3β1 or α6β4, InFs and InFs/β4 cells were treated with blocking antibodies against integrin β1, β4, or laminin-332, and their caspase-3 activity was compared. Inhibition of integrin β1 significantly inhibited caspase-3 activity in InFs and InFs/β4, whereas inhibition of integrin β4 and laminin-332 had no effect.

Similar to these results, induction of integrin β4 also reduced caspase-3 activity (Figure 4B). To show further that the caspase-3 activity of fibroblasts was decreased by laminin-332 upregulation, CAFs (Ln-332-low) and CAFs/β4 were treated with 10 μ*M *purified laminin-332, and the resulting caspase-3 activities were measured. Treatment with laminin-332 significantly decreased caspase-3 activity in both CAFs and CAFs/β4 (Figure 4C).

### Enhancement of anoikis resistance by integrin β4 neoexpression is mediated by Rac1 activation

**Figure 5:**
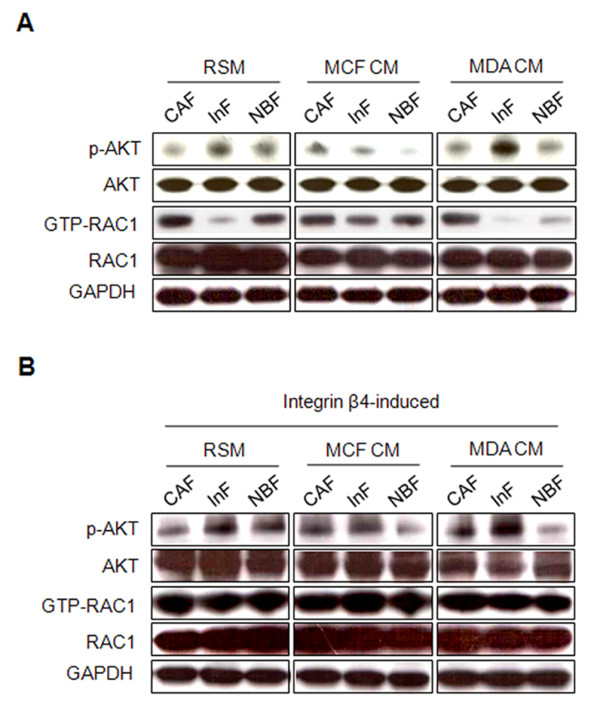
**Integrin β4 neoexpression induces Rac1 activation**. Akt phosphorylation and Rac1 activation is shown in **(A) **wild-type and **(B) **integrin β4-expressing fibroblasts. Wild-type and integrin β4-expressing fibroblasts were incubated in RSM, MCF7 CM, or MDA-MB-231 CM under anoikis conditions for 24 hours. Suspended fibroblasts were harvested for Rac1 pull-down assays.

Akt phosphorylation and Rac1 activation are associated with integrin-mediated anoikis resistance. In the absence of stimulation from cancer cells, InFs showed a higher level of phosphorylation of Akt (Ser473) than did CAFs and NBFs. Akt phosphorylation was enhanced by MDA-MB-231 CM and decreased by MCF7 CM (Figure 5A). GTP-bound Rac1 was downregulated in InFs but upregulated in CAFs and NBFs, and MCF7 CM further increased GTP-bound Rac1 in InFs (Figure 5A). Induction of integrin β4 expression dramatically increased GTP-bound Rac1, independent of stimulation from cancer cells, but did not have a significant effect on Akt phosphorylation (Figure 5B).

### Integrin β4 neoexpression suppresses aggregation of fibroblasts under anoikis conditions

**Figure 6:**
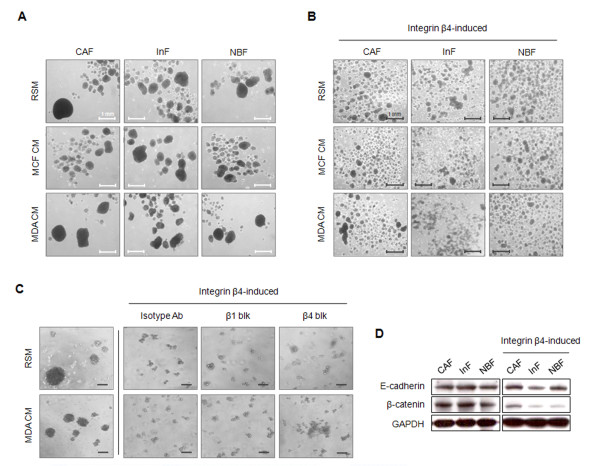
**Integrin β4 neoexpression suppresses fibroblast aggregation under anoikis conditions**. Formation of fibroblast aggregates under anoikis conditions in **(A) **the absence or **(B) **the presence of integrin β4. Fibroblasts (5 × 10^5^) were plated with RSM, MCF7 CM, or MDA-MB-231 CM on poly-HEMA-coated six-well plates and incubated for 24 hours. The formation of cell aggregates was observed with phase microscopy. **(C) **The scattering of integrin β4-expressing fibroblasts under anoikis conditions was not affected by blocking the laminin-332/integrin interaction. Fibroblasts (2.5 × 10^4^) were plated with RSM, MCF7 CM, or MDA-MB-231 CM on poly-HEMA-coated 96-well plates with blocking antibodies against integrin β1 or β4 (diluted 1:100 with RSM) or MDA-MB-231 CM for 24 hours. **(D) **Decreased expression of β-catenin may cause reduced fibroblast aggregation. Suspended cells were collected, lysed, and subjected to Western blot analysis of β-catenin expression.

Synoikis, the formation of cell aggregates in nonadherent conditions, has been proposed as a mechanism by which cells overcome anoikis. Therefore, we investigated the role of synoikis in the anoikis resistance of fibroblasts and whether it is affected by laminin-332 upregulation or integrin β4 neoexpression. In the absence of integrin β4 expression, the fibroblasts aggregated and formed big clusters under anoikis conditions; this aggregation was not affected by the upregulation of laminin-332 by MDA-MB-231 CM (Figure 6A). In contrast, integrin β4 expression suppressed fibroblast aggregation independent of laminin-332 (Figure 6B). To confirm whether fibroblast aggregation is caused by integrin β4 expression, rather than binding of laminin-332 to integrins, we treated fibroblasts with blocking antibodies against integrin β1 or β4. Neither blocking antibody affected fibroblast aggregation (Figure 6C). Based on these findings, we suggest that integrin β4 suppresses fibroblast aggregation in anoikis conditions. Synoikis is known to depend on E-cadherin-mediated cell-cell adhesion; therefore, the suppressive effect of integrin β4 expression on fibroblast aggregation may be caused by a decrease in E-cadherin expression. However, the fibroblasts expressing integrin β4 did not show decreased E-cadherin expression compared with wild-type controls. Because β-catenin displays E-cadherin on the cell surface, we postulated that downregulation of β-catenin may be the cause of defective E-cadherin-mediated cell-cell interaction. Consistent with this notion, β-catenin expression was decreased in fibroblasts expressing integrin β4 (Figure 6D).

## Discussion

In the present work, we hypothesized that myofibroblasts gain an anoikis-resistant phenotype during tissue remodeling through laminin-332 upregulation and integrin β4 neoexpression induced by interaction with invasive breast cancer cells. To prove our hypothesis, we isolated three types of fibroblasts, CAFs from the tumor zone (the epicenter of tumor burden), InFs from the interface zone (fibrosis), and NBFs from the normal zone (distal normal tissue) of IDC, and co-cultured them with tumor cells to investigate the tumor-stroma interaction. InFs, the myofibroblasts undergoing tissue remodeling, showed a higher resistance to anoikis than did CAFs and NBFs as a result of their high endogenous expression level of laminin-332 and its dramatic upregulation by conditioned medium from invasive MDA-MB-231 cancer cells. In addition, integrin β4 neoexpression was induced in fibroblasts by cell-to-cell contact with MDA-MB-231 cells regardless of the type of fibroblast, and expression of integrin β4 increased anoikis resistance to a level approximately twofold higher than that induced by laminin-332 upregulation. From these results, we conclude that invasive breast cancer cells confer an anoikis-resistant phenotype on myofibroblasts during tissue remodeling by inducing laminin-332 upregulation and integrin β4 neoexpression, and that InFs may be the primary myofibroblasts involved in tissue remodeling during invasion of breast cancer cells.

This may be the first study to describe how myofibroblasts acquire an anoikis-resistant phenotype during tissue remodeling by using a model of invasive breast cancer. Previously, one group reported that transforming growth factor β1 (TGF-β1) confers an anoikis-resistant phenotype on normal primary human fetal lung fibroblasts [25], and another group showed that the matricellular protein TSP1 induces anoikis resistance in mouse embryonic fibroblasts [21]. However, the fibroblasts used in these studies were not derived from tumor stroma, and we believed that it was necessary to investigate the development of anoikis-resistant myofibroblasts in the context of tumor progression. Therefore, in this study, we used IDC to examine the development of anoikis-resistant myofibroblasts during tissue remodeling.

We chose to study IDC for two reasons: the first was the dense fibrosis around the tumor burden, which is a phenotype of vigorous tissue remodeling [7-9], and the second was aberrant upregulation of laminin-332 in the fibrosis (in contrast, laminin-332 upregulation was not detected in its counterpart, DCIS) [13]. Because laminin-332 is widely known to promote cell survival, the anoikis resistance of myofibroblasts is likely mediated by laminin-332-dependent signaling. To test this assumption, we first investigated laminin-322 expression in CAFs, InFs, and NBFs and found that the expression pattern in the fibroblasts was the same as that *in situ *(Figure 1A). Based on α-SMA expression and their location in the tissue, CAFs and InFs are presumed to be the myofibroblasts that interact with invasive tumor cells during tissue remodeling.

Within the interface zone (IZ) adjacent to the tumor zone (TZ), invasive breast cancer cells may interact with InFs via cell-to-cell contact at the border between the TZ and IZ or with diffusible factors away from the border and within the IZ. It is therefore possible that the expression of laminin-332 or its receptors, integrin α3β1 and α6β4, is affected differently according to the type of interaction. To test this notion, fibroblasts were co-cultured with tumor cells to simulate cell-to-cell contact or were stimulated with the CM from tumor cells to test the role of diffusible factors. In our previous study, laminin-332 upregulation in the IZ was shown to be associated with tumor invasiveness [13]. Thus, MDA-MB-231 and MCF7 were used as invasive and noninvasive breast cancer cell types, respectively. After treatment with MDA-MB-231 CM, but not MCF7 CM, laminin-γ2, a subunit used to identify laminin-332, was upregulated in InFs, and this upregulation was maintained under anoikis conditions. Expression of laminin-γ2 in CAFs and NBFs was also promoted by MDA-MB-231 CM, but to a lesser extent than in InFs (Figure 1C). The degree of laminin-γ2 induction by diffusible factors produced by MDA-MB-231 seemed to depend on the endogenous capacity of fibroblasts to express laminin-γ2. According to previous reports, TGF-β is a major soluble factor produced by MDA-MB-231 [26-28]. Therefore, we examined whether laminin-γ2 upregulation by MDA-MB-231 CM is induced by TGF-β and found that the TGF-β inhibitor LY2157299 effectively suppressed laminin-γ2 expression (see Additional file 1, Figure S1). Based on this result, we suggest that expression of laminin-322 is at least partially induced by TGF-β secreted by invasive breast cancer cells. However, further evaluation is required because we did not test other cytokines that are highly secreted from invasive breast cancer cells. In contrast to laminin-332 upregulation by MDA-MB-231 CM, integrins α3, α6, and β1 were highly expressed in fibroblasts and not affected by either diffusible factors or direct contact with MDA-MB-231 cells (Figure 2, and see Additional file 1, Figure S3). Integrin β4 was not endogenously expressed in fibroblasts, but its expression was newly induced by direct contact with MDA-MB-231 cells. Almost no difference was found in the induction of anoikis resistance by integrin β4 neoexpression in the different types of fibroblasts.

When in direct contact with invasive breast cancer cells, the anoikis resistance of InFs involved both integrin α3β1 and integrin α6β4 as a result of integrin β4 neoexpression. Although both integrins are known receptors for laminin-332, their signaling pathways, signal duration, and dependency on laminin-332 are different. In the case of integrin α3β1, signaling was dependent on laminin-332 binding and mediated by Akt (Ser473) phosphorylation (Figure 5A). In contrast, integrin α6β4-mediated signaling was independent of laminin-332 and mediated through Rac1 activation (Figure 5B). Anoikis resistance induced by integrin α6β4 was approximately twofold higher than that induced by integrin α3β1 (Figure 3D). This is supported by a report that Rac1 activation through integrin α6β4 produces a long-lasting survival signal [18]. Furthermore, the long cytoplasmic tail of integrin β4 can be phosphorylated by other factors such as c-Met, independent of laminin-332 [29]. In addition to anoikis resistance, integrin β4 neoexpression suppressed fibroblast aggregation under anoikis conditions, possibly as a result of downregulation of β-catenin (Figure 6D). Many cells overcome anoikis through synoikis, the formation of cell clusters via E-cadherin-mediated interaction between cells. The expression of E-cadherin on the cell surface is known to be regulated by β-catenin [30]; therefore, decreased expression of β-catenin by integrin β4 neoexpression may cause defective surface expression of E-cadherin, which in turn suppresses fibroblast aggregation. Considering a recent report that myofibroblasts in the primary tumor site metastasize to the lung along with tumor cells [31], in the context of tumor metastasis, single cells or a small aggregate of myofibroblasts may be more likely to undergo distant migration with invasive tumor cells than might a large cluster.

**Figure 7:**
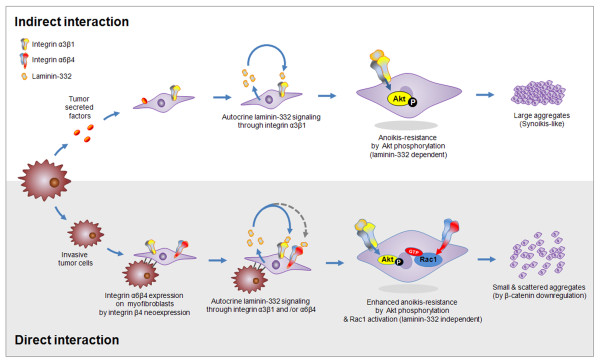
**Two possible mechanisms by which myofibroblasts acquire anoikis resistance via integrin receptors for laminin-332 during tissue remodeling**. Direct and indirect interactions may occur between invasive tumor cells and myofibroblasts in the interface zone of invasive ductal carcinoma. Myofibroblasts within the interface zone could be stimulated to express laminin-332 by diffusible factors from invasive breast cancer cells. Binding of laminin-332 to integrin α3β1 on the myofibroblasts turns on a cell-survival signaling pathway mediated by Akt phosphorylation. In this case, myofibroblasts may simultaneously use synoikis to overcome anoikis. In addition, in the border between the tumor zone and the interface zone, invasive breast cancer cells can directly interact with myofibroblasts, leading to integrin β4 neoexpression. Once integrin β4 is induced, myofibroblasts use both integrin α3β1 and α6β4 as receptors for laminin-332, and become resistant to anoikis via Akt phosphorylation (laminin-332 dependent) and Rac1 activation (laminin-332 independent). Moreover, integrin β4 neoexpression may inhibit myofibroblast aggregation through downregulation of β-catenin.

In summary, two mechanisms seem to exist by which myofibroblasts acquire anoikis resistance through laminin-integrin signaling during tissue remodeling as a result of either direct or indirect interaction between invasive breast cancer cells and myofibroblasts in the fibrosis (or interface zone) (Figure 7). For indirect interaction, factor(s) secreted from invasive tumor cells stimulate myofibroblasts to overexpress laminin-332. Laminin-332 binds to integrin α3β1 on myofibroblasts and turns on an autocrine cell survival signal mediated by Akt (Ser473) phosphorylation. In addition, myofibroblasts that lose their attachment spontaneously use synoikis to overcome anoikis.

Second, direct contact between invasive breast cancer cells and myofibroblasts induces integrin β4 neoexpression in the fibroblasts. Consequently, myofibroblasts express both integrin α3β1 and integrin α6β4 as receptors for laminin-332. In this case, cell-survival signals are mediated by Rac1 activation and Akt (Ser473) phosphorylation. Akt (Ser473) phosphorylation requires binding of laminin-332 to integrin α3β1, whereas Rac1 activation by integrin α6β4 is not dependent on laminin-332. Furthermore, integrin β4 neoexpression suppresses fibroblast aggregation under anoikis conditions, possibly through downregulation of β-catenin.

## Conclusions

Invasive breast cancer cells confer an anoikis-resistant phenotype on myofibroblasts during tissue remodeling by inducing laminin-332 upregulation and integrin β4 neoexpression. Our findings indicate that interface fibroblasts may be the primary myofibroblasts involved in tissue remodeling during breast cancer invasion.

## Abbreviations

CAF: cancer-associated fibroblast (fibroblasts isolated from the TZ); CMFDA: (5-chloromethylfluorescein diacetate); DCIS: ductal carcinoma *in situ*; ECM: extracellular matrix; IDC: invasive ductal carcinoma; InF: interface fibroblast (fibroblasts isolated from the IZ); IZ: interface zone (tumor-free fibrotic zone adjacent to tumor burden); Ln-332: laminin-332; NBF: normal breast fibroblast (fibroblasts isolated from the NZ); NZ: normal zone (normal tissue neighboring the IZ); Poly-HEMA: poly(2-hydroxyethyl methacrylate); TZ: tumor zone (tumor burden of IDC).

## Competing interests

We declare no conflicts of interest in all participants.

## Authors' contributions

BGK developed the concept and design of the study, performed the experiments, analyzed and organized the data, and wrote the manuscript. MQG carried out the isolation of fibroblasts, maintained them for subsequent experiments, and helped draft the manuscript. YPC performed cell sorting after co-culture, and participated in the design of the study. SK was involved in the anoikis assay. HRP and KSK participated in immunoassay. NHC participated in the selection of patient breast tissue material, the conception, design and coordination of the study, and helped draft the manuscript. All authors read and approved the final manuscript.

## Supplementary Material

Additional file 1 **Supplementary Figures 1, 2, 3, and 4**. Figure S1. TGF-β in MDA-MB-231 CM causes laminin-332 upregulation in InFs. Figure S2. Endogenous expression of integrins α3 and α6 in IDC fibroblasts. Figure S3. Expression of integrins β1 and β4 is not affected by diffusible factors from breast cancer cells. Figure S4. Exogenous expression of integrin β4 in IDC fibroblasts by transfection. Click here for file
