# Supplementary material for: Invasive breast cancer induces laminin-332 upregulation and integrin β4 neoexpression in myofibroblasts to confer an anoikis-resistant phenotype during tissue remodeling
Source: Breast Cancer Res. 2012 Jun 6;14(3):R88. doi: 10.1186/bcr3203 (PMC3446351; doi:10.1186/bcr3203)
Supplement: Additional file 1 — Supplementary Figures 1, 2, 3, and 4. Figure S1. TGF-β in MDA-MB-231 CM causes laminin-332 upregulation in InFs. Figure S2. Endogenous expression of integrins α3 and α6 in IDC fibroblasts. Figure S3. Expression of integrins β1 and β4 is not affected by diffusible factors from breast cancer cells. Figure S4. Exogenous expression of integrin β4 in IDC fibroblasts by transfection. [file bcr3203-S1.DOC]

**Supplementary Figures**


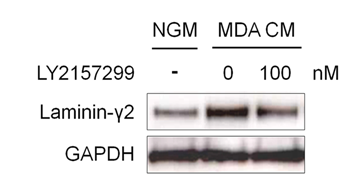


**Figure S1. TGFβ in MDA-MB-231 CM causes laminin-332 upregulation in InFs.** Confluent InFs on 100-mm dishes were cultured with NGM (normal growth media), MDA-MB-231 CM, or MDA-MB-231 CM with 100 nM LY2157299 for 24 h.


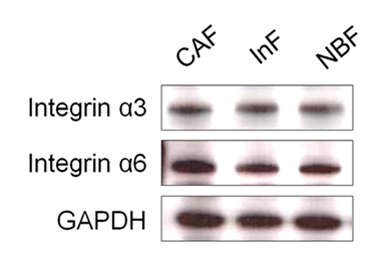


**Figure S2. Endogenous expression of integrin α3 and α6 in IDC fibroblasts.** CAFs, InFs, and NBFs were cultured in normal growth media for 24h.


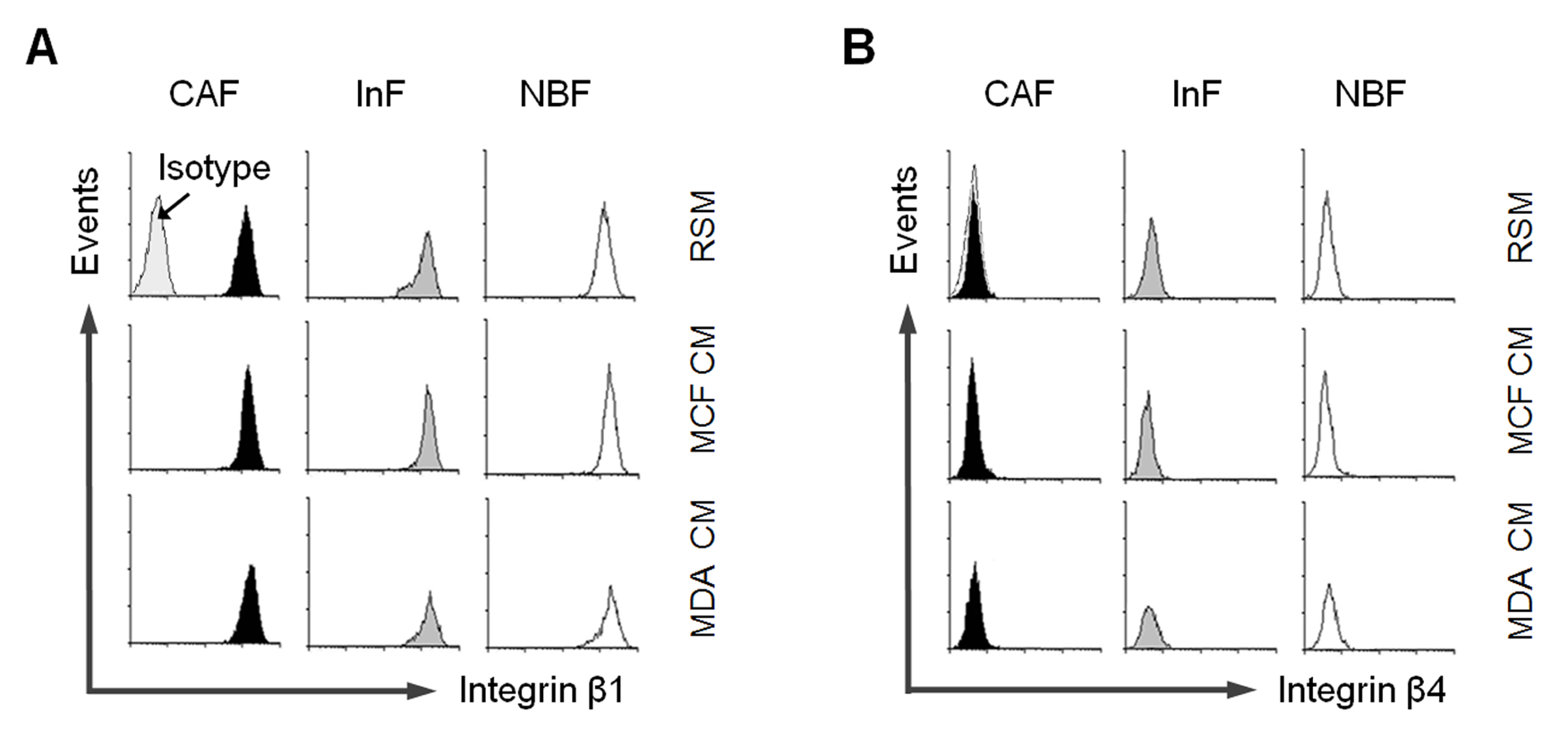


**Figure S3. Expression of integrin β1 and β4 is not affected by diffusible factors from breast cancer cells.** A) Integrin β1 and B) integrin β4 expression in fibroblasts stimulated with MCF7 CM or MDA-MB-231 CM. Fibroblasts were cultured in RSM, MCF7 CM, or MDA-MB-231 CM for 72 h, collected, and stained with PE-conjugated anti-integrin β1 or β4 antibodies.


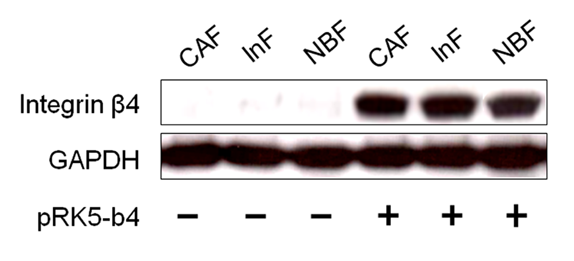


**Figure S4. Exogenous expression of integrin β4 in IDC fibroblasts by transfection.** For transient transfection of integrin β4, fibroblasts (5 × 10^5^ cells/ well) were plated on a 6-well plate and then transfected with 3 μg pRK6 β4 plasmid DNA and 3 μl MATRA in a total volume of 200 μl Opti-MEM on a Universal Magnet Plate for 15 min. The negative control was transfected with 3 μg pRK5.
